# Chemical Characterization and Antioxidant Potential of the Rowan (*Sorbus aucuparia* L.) Fruits from Alpine-Dinaric Region of Croatia^§^

**DOI:** 10.17113/ftb.61.04.23.8225

**Published:** 2023-12

**Authors:** Antonija Sulimanec, Karla Kragić, Ankica Sekovanić, Jasna Jurasović, Ines Panjkota Krbavčić, Nada Vahčić, Antonio Vidaković, Igor Poljak, Ivana Rumora Samarin

**Affiliations:** 1 Institute for Medical Research and Occupational Health, Ksaverska cesta 2, 10000 Zagreb, Croatia; 2 University of Zagreb Faculty of Food Technology and Biotechnology, Pierottijeva 6, 10000 Zagreb, Croatia; 3 University of Zagreb Faculty of Forestry and Wood Technology, Institute of Forest Genetics, Dendrology and Botany, Svetošimunska cesta 23, 10000 Zagreb, Croatia

**Keywords:** chemical characterization, macro- and trace elements, phenolic content, rowan, wild fruits

## Abstract

**Research background:**

The rowan (*Sorbus aucuparia* L.) is a small tree in the Rosaceae family with characteristic orange-red fruits. The raw fruits can be used for making jams, juices and puree, while the dried fruits are used for teas. In folk medicine, they have been used to prevent scurvy and bleeding or as a diuretic and laxative. The aim of this study is to characterize the proximate chemical composition, antioxidant potential and macro- and trace elements of the rowan fruits for their potential use as a functional food.

**Experimental approach:**

The fruits were collected from 12 populations in the Alpine-Dinaric region of Croatia. After collection, the samples were transported to the laboratory, cut into small pieces, placed in plastic containers and stored at -20 °C until analysis. Proximate chemical composition, including ash, water, cellulose, crude fat and crude protein, was determined according to standard methods and total carbohydrates as non-structural carbohydrates. Total phenolic content (TPC) and antioxidant capacity (TAC) were also measured. For multielement analysis, fruits were cleaned from the dust, lyophilised, homogenised and acid-digested in a microwave system. Concentrations of elements were determined using the inductively coupled plasma mass spectrometry.

**Results and conclusions:**

The basic constituents in the analysed fruits were (in %): water 76.53, total carbohydrates 17.45, crude proteins 2.98, crude fats 1.49, cellulose 1.07 and ash 1.29. On average, the TPC was 932 mg/100 g and the TAC was (60.1±14.5) % and (4.1±1.2) mmol/100 g, determined by DPPH and FRAP assay, respectively. Mass fractions of elements decreased as follows (in mg/kg): K 2485>Ca 459>P 206, Mg 193>Na 6.29>Fe 3.68>Mn 3.58>Zn 1.11>Cu 0.731>Mo 0.098>Co 0.003>Se 0.001. Compared to the literature, the phenolic and element content of the rowan fruits is similar to that of blueberry and raspberry. The obtained results suggest that rowan fruits have valuable nutritional properties and could be useful for fortification in the food industry.

**Novelty and scientific contribution:**

The importance of the obtained results is reflected in filling in the gaps in the literature on the composition of elements, especially on the content of essential macro- and trace elements as well as the antioxidant potential of rowan fruits.

## INTRODUCTION

The rowan (*Sorbus aucuparia* L., Rosaceae), commonly known as mountain ash, is a small deciduous tree naturally widespread in Europe, from extreme northern regions (Iceland, Fennoscandia, and Russia) to southern Europe (Iberian, Apennine, and Balkan Peninsula), where it is found only at higher elevations, while eastwards the species extends into Asia Minor and Northern Asia (*1*, *2*). It is well adapted to a short growing season and can also tolerate high summer temperatures, but high water stress has a negative effect, *i.e*. limiting factors for its spread are a combination of poor drought tolerance, adaptation to a short growing season and a need for cold rather than high temperatures for bud burst (*2*). It is characterized by pinnate leaves and bright orange-red, subglobose fruits with a diameter of 8–10 mm, *i.e.* pomes. The fruits remain on the tree during the winter and therefore provide food for birds, which disperse the seeds.

The fruits are typically processed into jams, jellies, juices or purees, rather than consumed fresh because of their specific astringent tannin-related taste and high levels of toxic parasorbic acid (*3*, *4*). In some European countries, such as Estonia, rowan tree fruits are commonly used as bread ingredient (*5*). In addition, alcoholic drinks are often made from or flavoured with rowan fruits, as the biochemical compounds in these fruits help to clear and preserve alcoholic drinks, simultaneously enriching them with flavour, astringency, bitterness and sugars (*6*). Owing to many nutritive and medicinal benefits, rowan fruits have been used in folk medicine as a remedy to prevent scurvy and bleeding, to treat intestinal obstructions, alleviate rheumatism and kidney diseases and as an antidiabetic agent (*7*-*9*). These beneficial effects may be attributed to various bioactive compounds, such as phenolic acids, flavonoids, vitamins and macro- and trace elements. Like most berries, rowan fruits also have a particularly high antioxidant capacity (*7*, *10*). The most abundant phenolic compounds in rowan fruit are quercetin dihexosides (78 % total flavonoids) and chlorogenic acid (33 to 73 % total hydoxycinnamic acid derivatives) (*11*, *12*). Moreover, rowan fruits are rich in carotenoids, sorbitol and ascorbic acid, all of which contribute to their antioxidant activity (*13*). According to Nile and Park (*14*), popular berries such as blackberry (*Rubus* spp. L.), blueberry (*Vaccinium corymbosum* L.), raspberry (*R*. *idaeus* L.) and strawberry (*Fragaria* × *ananassa* (Duchesne ex Weston) Duchesne ex Rozier) are good dietary sources of both macro- and trace elements that play an essential role in maintaining human health. Macroelements such as phosphorus, calcium, potassium and magnesium affect water and electrolyte balance and are involved in bone formation and muscle contraction, while trace elements like iron, manganese, copper, zinc and selenium are vital for metabolic catalysis, hormone functions and are key components of antioxidative enzymes (*15*).

During the last decade, numerous studies have primarily focused on investigating the antioxidant potential and phytochemical content of the rowan and other *Sorbus* L. (*sensu lato*) species (reviewed by Sarv *et al.* (*7*)), but there are very limited data on their elementary composition (*16*, *17*). This study aims to address this gap by characterizing the proximate chemical composition with particular emphasis on the essential macro- and trace elements along with the antioxidant potential of the rowan fruits originating from the Alpine-Dinaric region in Croatia for their potential use as functional ingredients in the food industry.

## MATERIALS AND METHODS

### Samples

**Fig. 1:**
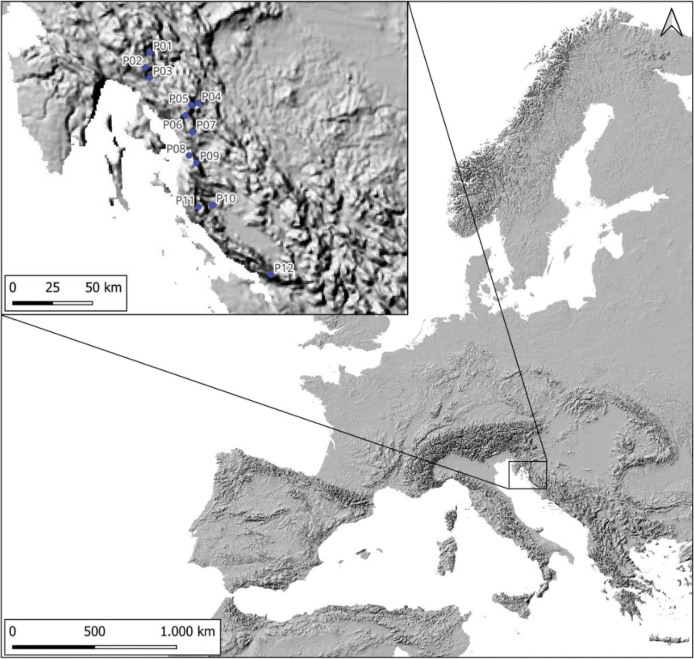
Locations of the 12 sampled *Sorbus aucuparia* populations. Sampling sites: P01=Crni Lug, P02=Risnjak, P03=Mrzla Vodica, P04=Jasenak, P05=Mala Javornica, P06=Duliba, P07=Miskovica, P08=Stolac, P09=Senjsko Bilo, P10=Jelovac, P11=Velebit, P12=Mali Alan

Fresh fruits of rowan trees were collected in the Alpine-Dinaric region of Croatia in 2020 (Fig. 1). A total of 12 rowan populations were included in the study. Fruits were manually picked from different plant specimens at the same location and packed in polyethylene bags (approx. 500 g each). The maturity was determined by full orange-red colouration. Overripe, dried or damaged berries were removed from the sample. The collected fruit samples were transported on ice to the University of Zagreb Faculty of Food Technology and Biotechnology, Croatia, for the proximate chemical composition and antioxidant profile analyses and to the Institute for Medical Research and Occupational Health in Zagreb, Croatia, for multielement analysis.

### Sample preparation and extraction of bioactive compounds

All fruits were cut into a pomace using a ProMix blender (HR2655/90; Philips, Shanghai, PR China) with the exception of those intended for multielement analysis, which were sliced with a ceramic knife. Each sample was placed in a plastic container, marked adequately and stored at -20 °C. For the determination of total phenols, antioxidant capacity (DPPH assay), and reducing power (FRAP assay) bioactive compounds were extracted as follows: approx. (6.00±0.01) g rowan pomace were added to 20 mL of extraction solvent (methanol (*φ*(MeOH)=2 %, *V*(MeOH):*V*(HCl)=95:5). The mixture, wrapped in aluminium foil to protect it from the light, was allowed to stand on a shaker for 60 min and then it was filtered through Whatman filter paper with 0.45 μm pore size using a Büchner funnel. The filtrate was adjusted to 50 mL in a volumetric flask using the extraction solvent. For multielement analysis, (10.00±0.01) g rowanberry pomace was freeze-dried at -50 °C for 72 h (HETOSIC; Heto Ltd., Gydevang, Denmark) and then homogenised with a Mixer Mill MM 400 (Retsch, Haan, Germany) at 29.5 Hz for 4 min using 2.8 mm ceramic balls and low density polyethylene (LDPE) sample vials. Freeze-dried and homogenised samples were stored at 4 °C until analysis.

### Determination of proximate chemical composition

Proximate chemical composition was determined using official AOAC methods: the ash content according to AOAC method 923.03 (*18*), water according to AOAC method 934.06 (*19*), crude protein trough transformation from the total nitrogen content by a conversion factor of 6.25 detected using the Kjeldahl method according to standard AOAC method 690.52 (*20*), and crude fat according to AOAC method 920.39 (*21*) using the Soxhlet apparatus (Inkolab, Zagreb, Croatia) and diethyl ether for 16 h. Cellulose was determined using AOAC method 973.18 (*22*). Total carbohydrate (TC) content was calculated as the difference using the following formula:

*w*(TC)= 100–(*w*(water)+ *w*(ash)+*w*(protein)+*w*(fat)) /1/

All analyses were performed in duplicate. Results for all methods are reported as mass fraction in %.

### Determination of total phenolic content

Total phenolic content (TPC) was determined by the photometric method using a Folin-Ciocalteu reagent (*23*) as follows: 0.04 mL diluted extract (1:10) was mixed with 3.16 mL distilled water (dH_2_O) and 0.2 mL Folin-Ciocalteu reagent and stirred well at vortex. The solution was left to rest for 3 min before a volume of 0.6 mL of a saturated sodium carbonate was added to the solution, vortexed again and incubated in a water bath (VK3EN; Inkolab) at 40 °C for 30 min. The absorbance was measured at 765 nm using a UV-1280 spectrophotometer (Shimadzu, Kyoto, Japan). TPC was calculated from the calibration curve with gallic acid as the standard. Results were expressed as mg of gallic acid equivalents (GAE) per 100 g of fresh fruits.

### Determination of antioxidant capacity

Antioxidant capacity (AC) was determined using the DPPH (2,2-diphenyl-2-picrylhydrazyl) method (*24*), which is based on the reduction of the stable DPPH radical in the presence of antioxidants with the change in colour from purple to yellow. In brief, 2 mL of diluted extract (1:10) were stirred with 2 mL of methanol and 1 mL of 0.5 mM DPPH methanolic solution, vortexed and kept in the dark at room temperature for 20 min. The absorbance was then measured at 517 nm using a UV-1280 spectrophotometer (Shimadzu) with methanol as a blank sample. The results for AC were calculated using the following equation:

AC=((*A*_control_ – *A*_sample_)/*A*_control_)·100 /2/

### Determination of the Fe(III) reducing antioxidant power

Fe(III) reducing antioxidant power (FRAP) assay was used to determine the antioxidant potential of the rowan fruit extract according to Benzie and Strain (*25*). The method is based on the reduction of the complex of Fe(III) and 2,4,6-tripyridin-2-yl-1,3,5-triazine (TPTZ) to the Fe(II) form at low pH with the change in colour from yellow to blue. The freshly prepared FRAP working solution (2.08 mL) was mixed with 0.240 mL of dH_2_O and 0.80 mL of diluted extract (1:10). The mixture was incubated at 37 °C for 5 min in a water bath (VK3EN; Inkolab) and then the absorbance was measured at 595 nm using a UV-1280 spectrophotometer (Shimadzu). The results were calculated according to the calibration curve for FeSO_4_·7H_2_O and expressed as mmol Fe(II) equivalents per 100 g of the fresh berries.

### Multielement analysis

Mass fractions of twelve essential macro- and trace elements (Na, Mg, P, K, Ca, Fe, Mn, Co, Cu, Zn, Se and Mo) were quantified using inductively coupled plasma mass spectrometry (ICP-MS) by Agilent 7500cx (Agilent Technologies, Tokyo, Japan). Before analysis, freeze-dried homogenised rowan fruit samples were digested in acid in a microwave system UltraCLAVE IV (Milestone, Sorisole, Italy). In brief, 0.250 g of each homogenate was digested in a 4-mL solution containing *φ*(HNO3)=50 % (65 % p.a. HNO_3_; Merck, Darmstadt, Germany) that had been purified by sub-boiling distillation duoPUR system (Milestone). After digestion, samples were adjusted to 6 g with ultrapure water and stored at 4 °C. The standard reference material, SRM 1570a spinach leaves (NIST, Gaithersburg, MD, USA), was used as a quality control material. All samples were prepared and digested in duplicate. Before ICP-MS analysis, samples were diluted 4-fold with a solution containing *φ*(HNO_3_)=1 % and 3 μg/L internal standards (germanium (Ge), rhodium (Rh), terbium (Tb), lutetium (Lu) and iridium (Ir)). Two replicates were prepared for each sample. The mass fractions were calculated according to the calibration curves for multielement standards prepared in 1 % HNO_3_ and were expressed in mg/kg on a fresh mass basis. Due to insufficient quantity of fruit samples from populations P09 (Senjsko bilo) and P10 (Jelovac), macro- and trace element analysis was not performed on these samples.

### Statistical analysis

Descriptive statistical parameters were calculated using the Statistica software package v. 13 (*26*). The results were shown as the arithmetic mean and standard deviations for chemical traits and for the studied populations. Assumptions of normality were checked using the Shapiro–Wilk test and the assumption of homogeneity of variance using Levene’s test. The procedure CRAN in R v. 3.2.2 (*27*) was used to calculate Spearman’s correlation coefficients between proximate chemical composition, antioxidant potential and mineral content (K, Ca, P, Mg, Na, Fe, Mn, Zn, Cu, Mo, Co and Se) detected in rowan fruits. The obtained values were interpreted according to Prion and Haerling (*28*), who defined results as: 0 to 0.20 negligible, 0.21 to 0.40 weak, 0.41 to 0.60 medium, 0.61 to 0.80 strong and ≥0.81 very strong. Statistical significance was set at 5 % (p<0.05). Statistically significant differences among the studied populations of rowan cultivars were determined using the analysis of variance (in the case of the homogeneity of variance) and Kruskal-Wallis test (in the case of the heterogeneity of variance). In addition, two principal component analyses (PCA) were performed to identify the divergence and structure of the studied populations: (*i*) for proximate chemical composition and antioxidant potential, and (*ii*) for macro- and trace elements. Biplots were constructed with two principal components showing the analysed populations and traits. The PC analyses were conducted using the MorphoTools, a set of R scripts in R v.3.2.2 (*27*) by following the manual of Koutecký (*29*).

## RESULTS AND DISCUSSION

Results of the average proximate chemical composition of all samples of rowan fruits are shown in Table 1. Differences between the studied populations were confirmed for all studied proximate constitutes, except for cellulose. Water is the main component (75.7 %) of the fruit, followed by total carbohydrates (17.4 %), crude proteins (3.0 %) and crude fats (1.5 %). The ash mass fraction was 1.1 %, while cellulose mass fraction was on average 1.3 %. The obtained results are in line with previously reported data (*1*, *30*, *31*). To the best of our knowledge, this is the first report that includes proximate chemical composition, macro- and trace elements as well as total phenolics and antioxidant capacity of rowan fruits. In addition, this is the first report on rowan fruit characteristics for this region. These fruits have been studied elsewhere, mainly from the forestry aspect and as a food source for frugivores (*30*, *32*). Looking at the results on individual population, the population with the highest water content was P10 (Jelovac), which consequently had the lowest total carbohydrate mass fraction. In contrast, the population with the lowest water content and the highest total carbohydrates was P06 (Duliba). The rowan fruits in population P12 (Mali Alan) were richest in crude protein, while the crude fat and cellulose mass fraction was highest in population P09 (Senjsko bilo). Population P04 (Jasenak) had the highest ash mass fraction.

**Table 1:** Descriptive statistical parameters and levels of significance for proximate chemical composition and antioxidant potential of rowan fruits from the Alpine-Dinaric region in Croatia

Population ID	*w*/%						*w*(phenols as GAE)/(mg/100 g)	*b*(FRAP)/(mmol/ 100 g)	DPPH scavenging activity/%
	Water	Ash	Fat	Protein	Total carbohydrates	Cellulose			
P01	79.0±1.6	1.0±0.4	1.0±0.1	2.9±0.3	15.0±1.8	1.09±0.2	933±61	2.9±1.6	48.4±5.3
P02	75.3±3.9	1.08±0.3	1.2±0.4	3.2±0.6	17.9±4.5	1.42±0.5	1077±193	5.8±1.0	56.6±3.3
P03	77.3±2.9	1.0±0.4	1.3±0.4	3.2±0.6	16.0±3.5	1.24±0.09	942±100	4.3±0.8	49.9±3.9
P04	79.4±2.6	1.6±0.5	1.6±0.2	3.2±0.1	12.7±1.4	1.43±0.6	679±93	5.6±1.5	50.2±4.7
P05	79.6±1.8	1.16±0.3	1.5±0.6	3.2±0.4	13.3±2.2	1.29±0.2	837±86	4.5±0.7	87.7±4.1
P06	68.2±3.8	1.3±0.6	1.3±0.3	2.1±0.3	26.0±3.5	1.07±0.5	894±135	3.8±0.4	54.7±4.4
P07	78.2±3.4	0.8±0.1	1.7±0.8	3.1±0.4	15.3±3.9	1.01±0.2	669±84	3.4±0.3	61.7±2.4
P08	76.7±1.2	0.8±0.2	1.2±0.4	3.0±0.6	17.1±1.8	1.22±0.4	824±129	3.8±0.4	86.4±3.6
P09	76.3±1.7	1.3±0.0	2.4±0.4	3.3±0.3	15.0±1.5	1.75±0.4	926±185	5.5±1.5	52.1±2.4
P10	80.7±2.7	0.8±0.2	2.2±0.9	2.9±0.1	11.9±1.6	1.51±0.3	758±80	3.4±0.4	60.8±1.7
P11	73.4±3.4	0.8±0.1	1.4±0.6	3.0±0.2	20.3±4.0	1.10±0.2	1185±365	3.4±0.3	49.7±1.9
P12	74.2±3.5	1.0±0.3	1.4±0.3	3.5±0.3	18.2±3.0	1.39±0.4	1265±892	3.4±0.5	53.6±4.4
Total	75.7±4.6	1.1±0.4	1.5±0.6	3.0±0.6	17.5±5.0	1.3±0.4	932±330	4.1±1.2	60.1±14.5
p-value	<0.01	<0.05	<0.01	<0.01	<0.01	n.s.	<0.01	<0.01	<0.01

Results are presented as the arithmetic mean (M) and standard deviation (S.D.). Sampling sites: P01=Crni Lug, P02=Risnjak, P03=Mrzla Vodica, P04=Jasenak, P05=Mala Javornica, P06=Duliba, P07=Miskovica, P08=Stolac, P09=Senjsko Bilo, P10=Jelovac, P11=Velebit, P12=Mali Alan. Results are presented as mean value±standard deviation, *N*=63

Results are presented as mean value±standard deviation, *N*=63

Results of the total phenolic content (TPC), antioxidant capacity (DPPH) and reducing power (FRAP) of the rowan fruits are given in Table 1. On average, TPC mean values were 932 mg/100 g, antioxidant capacity as percentage of residual DPPH was 60.14 %. The observed TPC in rowan fruits from the mountain region in Croatia was 2-fold higher than the TPC of 427 mg/100 g reported in rowan fruits in the Czech Republic (*3*) and of 520 mg/100 g in Finland (*33*), and similar to the TPC in the ‘Granatnaja’ cultivar (733–958 mg/100 g) (*3*, *34*). Similar results were obtained for cultivars from Montenegro and Serbia with TPC, expressed as GAE, ranging from 5.25 to 15.91 g/kg (*35*) and for different cultivars in the Czech Republic ranging from 8.81 to 16.31 g/kg (*36*). The content of phenolic compounds in fruits is determined by various biological and environmental factors and by conditions of food processing such as time of harvesting and storage. For example, it was shown that wild fruits from cold climate and a short vegetation season had a higher TPC than those that grow in a milder climate (*37*). Our results on TPC in rowan fruits that grow in the mountain region were similar to the TPC found in bilberry (1040 mg/100 g) and blackberry (980 mg/100 g) (*38*), which are recognised as fruits with particularly high TPC content (*37*) and slightly higher than the TPC found in different cultivars of raspberry (278–714 mg/100 g) (*39*). Reducing power expressed as molal concentration of Fe(II) of the rowan fruits in our study was on average 4.11 mmol/100 g, which is similar to the results obtained by Hukkanen *et al.* (*34*), where reducing power ranged between 6 and 10.5 mmol/100 g.

Several studies have shown that rowan fruits can be a good source of different bioactive components (*7*, *35*) and as such have a potentially positive effect on human health. In addition to the degree of maturity, it is very important to monitor the harvesting location of the fruit, because it is known that different phenolic compounds can be under the influence of different environmental factors (*40*). Therefore, the results of this study could be used to evaluate better locations for cultivation with the desired bioactive components. Accordingly, at the level of individual population, the highest phenolic content was found in populations P12 (Mali Alan), P11 (Velebit) and P02 (Risnjak), which are the three highest located populations in our study. Higher elevation has been previously reported to have beneficial effect on phenolic content in rabbiteye blueberry (*Vaccinium ashei* J.M.Reade ‘Brightwell’) (*41*) and Andean blueberry (*V*. *floribundum* Kunth) (*42*). FRAP was highest in P02 (Risnjak), P04 (Jasenak) and P09 (Senjsko bilo), while antioxidant activity was highest in fruits from populations P05 (Mala Javornica) and P08 (Stolac).

The average mass fractions of essential elements determined in the rowan fruits are given in Table 2. For macroelements, the highest mass fraction was observed of K (2485 mg/kg), followed by Ca (459 mg/kg), P (206 mg/kg), Mg (193 mg/kg) and Na (6.3 mg/kg). The mass fractions of trace elements decreased as follows: Fe (3.7 mg/kg), Mn (3.6 mg/kg), Zn (1.1 mg/kg), Cu (0.7 mg/kg) and Mo (0.10 mg/kg), while the mass fractions of Co and S were lower than 0.01 mg/kg. Comparing our results with the study by Aslantaş *et al.* (*16*), in which the composition of elements of wild fruits from Anatolia in Turkey was characterized, there were notable differences in the mass fractions of trace elements, particularly of Fe (3.7 *vs* 24.2 mg/kg), Zn (1.1 *vs* 8.6 mg/kg) and Cu (0.7 *vs* 2.9 mg/kg). These differences could be attributed to differences in industrial air pollution and soil properties between Croatia and Turkey. To characterize the route of element exposure (mineral dusts *versus* plant uptake from the soil), Shotyk (*43*) compared the amounts of various trace elements in fruits of eight different plant species with lithophilic elements (*e.g*. Al). The results showed that the content of Fe in wild fruits depended on atmospheric dust, suggesting that the Fe in these fruits originates mainly from mineral dust deposition. On the other hand, the amounts of Zn and Cu in the fruits were found to be highly affected by plant uptake through the roots, suggesting that these elements are predominantly taken up by the plants from the soil. Regarding the elemental composition among different populations in our study, the rowan fruits of population P02 (Risnjak) had the highest macro- and trace element content with above-average values for 10 out of 12 analysed elements, followed by populations P12 (Mali Alan) and P11 (Velebit) with seven and six above-average values, respectively (Table S1). On the other hand, populations P04 (Jasenak) and P07 (Miškovica), followed by P05 (Mala Javornica), had the lowest elements content in their fruits. The Kruskal-Wallis test revealed statistically significant differences between the studied populations for all analysed elements, except P and Cu.

**Table 2:** Limit of detection (LoD) for each element analysed by ICP-MS method and average element mass fraction in rowan fruits and in standard reference material

Element	LoD(element)/(mg/kg)	*w*(element)/(mg/kg)							Recovery/%	
Rowan fruits	SRM 1570a					
	Measured value			Certified value				
Na	6.12	6.3±3.1	18318±826		18210±230				101	
Mg	0.12	193±47	9154±424		9000				102	
P	3.80	206±65	5185±179		5187.000±0.007				100	
K	12.8	2485±460	28528±935		29000±260				98	
Ca	2.99	459±151	14544±564		15260.00±0.07				95	
Fe	0.763	3.7±0.9	324±10		nd				nd	
Mn	0.007	3.6±2.9	76.5±3.3		76.0±1.2				101	
Co	0.0003	0.003±0.001	0.37±0.02		0.39±0.03				95	
Cu	0.037	0.7±0.3	12.4±0.5		12.2±0.9				101	
Zn	0.059	1.1±0.4	82.3±2.3		82.3±3.9				100	
Se	0.001	<LoD–0.002	0.111±0.003		0.115±0.004				96	
Mo	0.002	0.10±0.06	0.37±0.02		nd				nd	

Resultsarepresentedasmeanvalue±standarddeviation.SRM1570a=standardreferencespinachleaves(NIST,Gaithersburg,MD,USA), nd=not detected

Due to limited literature data on macro- and trace elements content in rowan fruits, we compared our results with data reported for fruits of other plant species such as blackberry, blueberry, raspberry, strawberry, *etc*. Compared to the amounts of various bioactive components in commonly consumed fruits reviewed by Nile and Park (*14*), rowan fruits from the Croatian mountain region are similar to raspberries and blackberries in the composition of macroelements and to blueberries and black currant in trace elements. Potassium was most abundant element found in rowan fruits. This element is important for distribution of fluids inside and outside cells, regulation of acid-base balance and muscle contraction (*44*). Mass fractions of K in rowan fruits were about 2.5-fold higher than those reported by Pereira *et al*. (*45*) in blueberry, raspberry, strawberry and blackberry from Brazil (954 to 1594 mg/kg). The mass fraction of Ca in rowan was 1.5-fold higher than in other fruits (459 *vs* 283 mg/kg), while the mass fraction of Mg did not differ significantly (119 to 243 mg/kg). Rowan is most comparable to raspberry in macroelements, especially in the mass fractions of Mg (220 mg/kg) and P ((290 mg/kg) (*46*). For trace elements, rowan fruits have quite similar mass fractions of Fe, Zn and Mn to wild rosehip (*43*, *47*) and of Zn, Cu and Co to blueberry (*43*, *45*).

The Spearman’s correlation coefficients between proximate chemical composition, antioxidant potential and elements (K, Ca, P, Mg, Na, Fe, Mn, Zn, Cu, Mo, Co and Se) found in rowan fruits are shown in Table S2. Very strong negative correlations were found between water content and total carbohydrates, as well as between DPPH and Mo. On the other hand, very strong positive correlations were determined between K, Ca and Mg and between Mg and Fe. The largest number of statistically significant correlations was found for Mg, while crude fat content was not correlated to any of the analysed traits. Crude protein content, total carbohydrates, phenols, DPPH, P, Na, Co and Se were significantly correlated with only one other trait. Positive correlation was detected between P and Na, which is in accordance with the results for another similar fruit, Mediterranean buckthorn (*Rhamnus alaternus* L.) (*48*). One of the components with the highest number of significant correlations was Mg, which was previously reported for sweet chestnut (*Castanea sativa* Mill.) (*49*). In contrast to the results of previous studies on *Sorbus* fruits (*35*), no significant correlation was found between the total phenolic content and antioxidant activity determined by DPPH.

PC analysis based on proximate chemical composition and antioxidant potential revealed that the first three components had eigenvalues >1, 3.31, 2.11 and 1.42 respectively (Table 3). The first principal component (PC1) explained 36.72 % of the total variability, while the second principal component (PC2) explained 23.42 % of the total variability. High correlations were found between PC1 and total carbohydrates (-0.90), water (0.80) and cellulose (0.74), while for PC2 high correlation was found for ash (0.76).

**Table 3:** Pearson’s correlation coefficients between nine chemical traits and scores of the first three principal components (PC)

Trait	PC1	PC2	PC3
Water	0.797	-0.508	-0.054
Ash	0.182	0.758	0.416
Fat	0.651	0.157	0.202
Proteins	0.606	0.032	-0.713
Cellulose	0.737	0.512	-0.158
Total carbohydrates	-0.895	0.358	0.070
Total phenolic content	-0.447	0.353	-0.786
FRAP	0.521	0.657	0.124
DPPH	0.172	-0.551	0.182
Eigenvalue	3.31	2.11	1.42
Total variance/%	36.72	23.42	15.78

PC analysis of 12 macro- and trace elements revealed similar results, with PC1 explaining 55.36 % and PC2 explaining 16.35 % of the total variability (Table 4). The first three components had eigenvalues >1, with values of 6.64, 1.96 and 1.36, respectively. In addition, PC1 had strong negative correlations with Mg, Fe, Zn, K, Mn and Se, whereas PC2 demonstrated similarly strong negative correlation values with Mo and Na.

**Table 4:** Pearson’s correlation coefficients between 12 macro- and trace elements and scores of the first three principal components (PC)

Trait	PC1	PC2	PC3
K	-0.792	-0.004	-0.464
Ca	-0.618	-0.067	-0.597
P	-0.467	-0.752	0.110
Mg	-0.959	-0.018	-0.009
Na	-0.125	-0.870	0.185
Fe	-0.922	0.102	-0.021
Mn	-0.760	0.304	0.417
Zn	-0.863	0.176	0.286
Cu	-0.627	0.206	-0.115
Mo	0.067	-0.809	-0.175
Co	-0.588	-0.166	0.648
Se	-0.702	-0.059	-0.277
Eigenvalue	5.57	2.19	1.41
Total variance/%	36.72	23.42	15.78

**Fig. 2:**
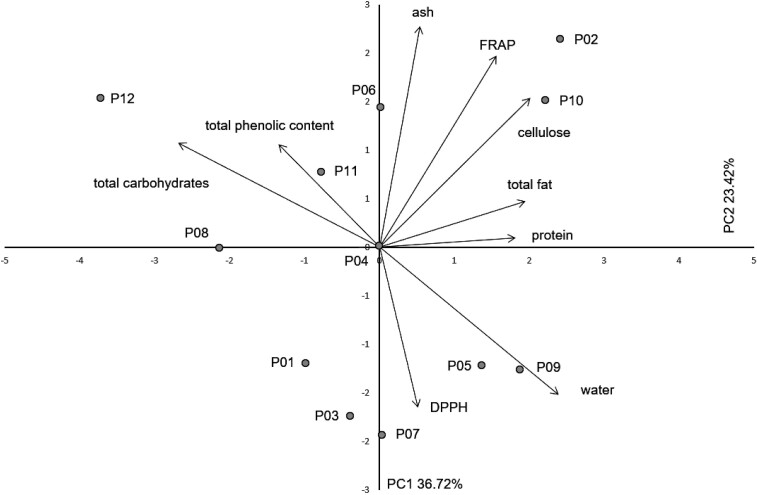
Biplot of the principal component (PC) analysis based on proximate chemical composition and antioxidant potential of 12 rowan populations from Alpine Dinaric region of Croatia. Sampling sites: P01=Crni Lug, P02=Risnjak, P03=Mrzla Vodica, P04=Jasenak, P05=Mala Javornica, P06=Duliba, P07=Miskovica, P08=Stolac, P09=Senjsko Bilo, P10=Jelovac, P11=Velebit, P12=Mali Alan

**Fig. 3:**
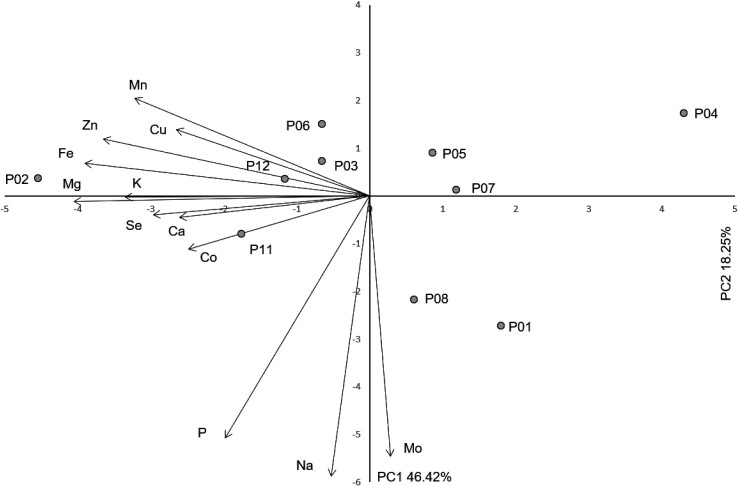
Biplot of the principal component (PC) analysis based on essential macro- and trace elements of 12 rowan populations from Alpine Dinaric region of Croatia. Sampling sites P01=Crni Lug, P02=Risnjak, P03=Mrzla Vodica, P04=Jasenak, P05=Mala Javornica, P06=Duliba, P07=Miskovica, P08=Stolac, P09=Senjsko Bilo, P10=Jelovac, P11=Velebit, P12=Mali Alan

Furthermore, none of the PC analyses (Fig. 2 and Fig. 3) revealed a substructure of the studied populations. As an insect-pollinated and animal-dispersed species, rowan pollen and seeds are able to spread in all directions and across potential, smaller barriers (*50*). The most important dispersal agents of rowan seeds are birds, especially of the genus *Turdus* L. (*51*). As there are no major geographical barriers between the populations included in this study, it is safe to assume that populations communicate very well despite the low population density, so there is no clear geographical pattern. In contrast, Poljak *et al.* (*52*) showed that populations of service tree (*Sorbus domestica* L.) are structured due to the environmental heterogeneity over the studied geographical area. A model-based population structure analysis using morphometric and chemical fruit characteristics revealed three eco-geographical groups of service tree populations (*52*). Similar results were reported for leaf morphometric analysis of service tree populations (*53*).

## CONCLUSIONS

In this study, the antioxidant properties and chemical composition of the fruits (both proximal and elemental) of 12 rowan populations from the Alpine-Dinaric region of Croatia were analysed. The obtained results are important due to limited data available on the elemental composition, especially on the content of essential macro- and trace elements and the antioxidant potential of rowan fruits. The results show that rowan fruits have a high antioxidant capacity and a good profile of essential elements, especially the macroelement potassium and the trace elements iron, manganese and zinc. Comparing the obtained results with the existing literature, rowan fruits have a similar phenolic and elemental content to blueberries and raspberries. The results of the study show that rowan fruits have great potential for the fortification of functional foods and dietary supplements. Their high antioxidant capacity and good profile of essential elements make them a valuable ingredient for the improvement of the nutritional and health benefits of various food products. This autochthonous, non-traditional fruit species needs to be further studied for its bioactive properties and its application in the industry should receive more attention.

## Appendix

**Table S1:** Arithmetic means on fresh mass basis for macro- and trace elements of rowan fruits from the Alpine-Dinaric region in Croatia

Population ID	*w*/(mg/kg)											
K	Ca	P	Mg	Na	Fe	Mn	Zn	Cu	Mo	Co	Se
P01	2362	409	242	161	9.14	2.66	1.04	0.786	0.584	0.168	0.002	0.001
P02	2738	498	225	236	5.37	4.90	9.91	1.897	0.767	0.093	0.006	0.002
P03	2273	422	189	200	7.45	3.70	6.06	1.226	0.725	0.026	0.003	0.001
P04	1581	391	98	119	2.53	2.72	2.45	0.625	0.442	0.086	0.002	0.000
P05	2242	296	190	174	4.66	3.30	2.94	1.419	0.824	0.076	0.002	0.001
P06	2599	450	170	211	2.56	3.41	3.29	1.101	0.895	0.071	0.003	0.001
P07	2201	396	224	165	4.61	3.59	1.78	0.840	0.581	0.057	0.002	0.001
P08	1887	374	237	190	8.66	3.40	2.46	0.981	0.691	0.149	0.005	0.001
P11	2642	593	201	204	5.99	4.41	3.19	1.210	0.682	0.147	0.002	0.002
P12	2978	491	208	213	5.74	3.95	2.93	1.095	0.960	0.073	0.002	0.001
Total	2485	459	206	193	6.29	3.68	3.58	1.113	0.731	0.098	0.003	0.001

Sampling sites: P01=Crni Lug, P02=Risnjak, P03=Mrzla Vodica, P04=Jasenak, P05=Mala Javornica, P06=Duliba, P07=Miskovica, P08=Stolac, P09=Senjsko Bilo, P10=Jelovac, P11=Velebit, P12=Mali Alan

**Table S2:** Spearman’s correlation coefficients between proximate chemical composition, antioxidant potential and elements (K, Ca, P, Mg, Na, Fe, Mn, Zn, Cu, Mo, Co and Se) detected in rowan fruits

	Ash	Fat	Protein	TCarb	Cellul	Phenols	FRAP	DPPH	K	Ca	P	Mg	Na	Fe	Mn	Zn	Cu	Mo	Co	Se
Water	-0.321	-0.006	0.018	**-0.927**	-0.067	-0.491	-0.006	0.224	-0.442	-0.527	-0.055	**-0.673**	-0.188	-0.564	-0.321	-0.091	-0.406	-0.067	-0.552	-0.067
Ash		0.103	0.164	0.079	0.418	0.200	0.406	-0.030	**0.745**	0.467	0.018	**0.636**	-0.200	0.321	0.333	0.370	**0.685**	0.164	0.030	0.091
Fat			0.370	-0.164	0.103	-0.248	0.127	0.212	-0.030	-0.067	0.273	0.224	-0.200	0.455	0.176	0.406	0.018	-0.006	0.358	0.127
Protein				-0.152	**0.782**	0.430	0.345	-0.006	-0.006	0.261	-0.345	0.139	-0.491	0.285	0.406	0.309	-0.200	0.152	0.164	0.503
TCarb					-0.152	0.430	-0.127	-0.127	0.224	0.358	-0.103	0.430	0.152	0.382	0.127	-0.127	0.261	-0.079	0.382	-0.091
Cellul						0.430	**0.648**	0.055	0.273	0.345	-0.370	0.442	-0.285	0.285	**0.745**	0.624	0.285	0.127	0.285	0.552
Phenols							-0.091	-0.624	0.055	0.224	0.006	0.103	0.139	-0.067	0.030	-0.164	-0.091	**0.661**	0.067	0.127
FRAP								0.382	0.079	-0.018	-0.564	0.394	-0.624	0.127	**0.636**	0.491	0.539	-0.406	0.418	0.055
DPPH									0.055	-0.067	-0.503	0.188	-0.370	0.261	0.406	0.527	0.418	**-0.830**	0.030	0.200
K										**0.855**	0.176	**0.818**	0.200	**0.673**	0.503	0.503	**0.636**	0.006	0.164	0.491
Ca											0.030	**0.733**	0.115	**0.782**	0.527	0.358	0.285	-0.018	0.285	**0.661**
P												0.042	**0.673**	0.042	-0.333	-0.091	-0.103	0.576	0.103	0.055
Mg													0.067	**0.830**	**0.782**	**0.697**	**0.770**	-0.164	**0.636**	0.503
Na														0,030	-0.079	0.055	0.006	0.479	-0.030	0.103
Fe															**0.685**	0.624	0.382	-0.261	0.576	0.600
Mn																**0.879**	0.600	-0.309	0.576	0.612
Zn																	**0.636**	-0.212	0.382	0.588
Cu																		-0.285	0.321	0.115
Mo																			-0.224	-0.067
Co																				0.358

Significant correlations (p<0.05) are marked in bold. TCarb=total carbohydrate, Cellul=cellulose
